# Prevalence, spatial distribution, and economic impact of bovine cysticercosis in slaughtered animals under federal inspection in Minas Gerais, Brazil (2021-2022)

**DOI:** 10.1590/S1984-29612026025

**Published:** 2026-07-20

**Authors:** Manoel Eduardo Silva, Felipe Boniedj Ventura Alvares, Thais Ferreira Feitosa, Marcela Zonta Rodrigues, Vinícius Longo Ribeiro Vilela

**Affiliations:** 1 Ministério da Agricultura e Pecuária – MAPA, Serviço de Inspeção Federal, Brasília, DF, Brasil; 2 Universidade Federal de Campina Grande - UFCG, Programa de Pós-graduação em Ciência e Saúde Animal, Patos, PB, Brasil; 3 Instituto Federal da Paraíba - IFPB, Departamento de Medicina Veterinária, Sousa, PB, Brasil; 4 Instituto Federal do Espírito Santo - IFES, Departamento de Tecnologia de Alimentos, Piúma, ES, Brasil

**Keywords:** Basic sanitation, Bos taurus, Cysticercus bovis, *Post mortem* inspection, Taenia saginata, Saneamento básico, Bos taurus, Cysticercus bovis, inspeção *post mortem*, Taenia saginata

## Abstract

This study evaluated prevalence, productive losses, and spatial distribution of cases in cattle slaughtered under the Federal Inspection Service (SIF) in Minas Gerais (2021–2022). A total of 297,802 animals were inspected; cysticercosis was detected in 0.44% (531/120,011) of cattle in 2021 and 0.24% (419/177,791) in 2022. Detection led to immediate condemnation of viscera, reducing carcass value by about 10%, and to conditional utilization of 37.1% (352/950) of carcasses by chilling and 15.8% (150/950) by heat treatment, with value reductions of 30% and 70%, respectively. Cysticerci were mainly located in the heart (45.2%), liver (40.1%), and head (21.2%), with rare occurrences in the tongue, diaphragm, and lungs. Estimated losses over two years exceeded R$ 900,000, likely underestimated given that SIF slaughter often supplies export markets. Spatial distribution analysis showed a higher concentration of positive animals originating from the municipalities of Nanuque, Governador Valadares, and Carlos Chagas. Despite the relatively low prevalence, the spatial concentration of cases and the observed distribution pattern suggest the influence of regional environmental factors in maintaining bovine cysticercosis transmission.

## Resumo

Este estudo avaliou a prevalência, as perdas produtivas e a distribuição espacial dos casos em bovinos abatidos sob o Serviço de Inspeção Federal (SIF) em Minas Gerais (2021–2022). Um total de 297.802 animais foi inspecionado; a cisticercose foi detectada em 0,44% (531/120.011) dos bovinos em 2021 e em 0,24% (419/177.791) em 2022. A detecção levou à condenação imediata das vísceras, reduzindo o valor da carcaça em cerca de 10%, e à utilização condicional de 37,1% (352/950) das carcaças por resfriamento e de 15,8% (150/950) por tratamento térmico, com reduções de valor de 30% e 70%, respectivamente. Os cisticercos localizaram-se principalmente no coração (45,2%), fígado (40,1%) e cabeça (21,2%), com ocorrências raras na língua, diafragma e pulmões. As perdas estimadas ao longo de dois anos excederam R$ 900.000, provavelmente subestimadas, uma vez que o abate sob SIF frequentemente abastece mercados de exportação. A análise espacial revelou aglomerados nos municípios de Nanuque, Governador Valadares e Carlos Chagas. Esse padrão pode estar parcialmente associado às bacias hidrográficas que recebem efluentes não tratados provenientes de municípios vizinhos com baixa cobertura de saneamento básico, como Jequitinhonha e Carlos Chagas, favorecendo a contaminação ambiental por ovos de *Taenia saginata*.

## Introduction

Bovine cysticercosis, caused by Cysticercus bovis, the larval form of *Taenia saginata* (Taylor, Coop, Wall, 2017), remains a relevant concern for Brazilian cattle production not only because of its zoonotic nature, with the risk of transmitting taeniasis to consumers of infected meat (CDC, 2022), but also due to the economic impact associated with carcass disposition, which frequently results in condemnations or conditional utilization (Camba & Alves, 2020). Although many regions present low prevalence rates under official inspection, the economic losses resulting from carcass condemnation or conditional use remain significant (Freitas et al., 2023). Studies in Minas Gerais, for example, have reported losses exceeding R$ 1 million over ten years (2005-2014) in slaughterhouses operating under sanitary inspection (Uberlândia-MG) due to bovine cysticercosis (Rezende et al., 2018).

In 2022, the Brazilian cattle herd was estimated at 202 million head. In the same year, approximately 42.3 million animals were slaughtered under official inspection services, resulting in 10.7 million tons of carcass equivalent (TEC). Of this total, 7.7 million TEC (72%) were consumed domestically, while 3 million TEC (28%) were exported (ABIEC, 2023). These figures highlight the importance of the cattle industry for the country and demonstrate that even low infection rates can have major economic impacts, particularly in large-scale production regions (Dutra et al., 2012).

Cattle, as intermediate hosts, develop cysticercosis after ingesting eggs or proglottids released into the environment. Major contamination sources include human feces deposited in pasture areas, farm workers infected with taeniasis who handle the herd, the presence of birds and flies, carrying eggs, and the use of untreated wastewater, sludge, or sewage in irrigation (Bucur et al., 2019; de Souza et al., 2025a).

Humans, the definitive hosts, acquire taeniasis by consuming raw or undercooked beef containing viable cysticerci. It is estimated that more than 77 million people worldwide are infected with this zoonotic parasite (Caixeta et al., 2022). A single infected person may release several proglottids daily, each containing 50,000 to 80,000 eggs, capable of contaminating extensive areas and generating outbreaks in cattle (Hoberg, 2002; de Souza et al., 2025a).

Thus, the occurrence of cysticercosis in herds is directly associated with the presence of human carriers, combined with poor hygiene conditions, inadequate sanitation, and limiting socioeconomic factors (Freitas et al., 2023). In Espírito Santo, Rossi et al. (2022) demonstrated that mesoregions with higher human population density and lower sanitation infrastructure exhibited higher prevalence of cysticercosis and taeniasis, showing that intermunicipal environmental vulnerability is a determining factor. In the western region of Minas Gerais, following the update of the Brazilian Industrial and Sanitary Inspection Regulation for Products of Animal Origin (RIISPOA) in 2017, there was a change in the destination of infected carcasses, although prevalence remained significant in some establishments (Freitas et al., 2023).

The taeniasis-cysticercosis complex constitutes an important public health issue in both urban and rural areas with sanitation deficiencies, favoring the maintenance of the parasite’s lifecycle (Duarte et al., 2016). This reality is observed in Latin America, Africa, the Mediterranean region, and throughout Brazil, where prevalence remains elevated (Braae et al., 2018). The joint committee of the Food and Agriculture Organization (FAO) and the World Health Organization (WHO) classified *T. saginata* as the third most relevant parasite for international trade (FAO, 2014). It is estimated that nearly 60 million people are infected worldwide, especially in livestock-producing regions. This importance derives not only from the direct impact on human and animal health but also from the economic losses during slaughter and the costs associated with control programs (Macpherson & Bidaisee, 2015).

In Brazil, bovine cysticercosis is the main parasitic disease diagnosed during post-mortem inspection and the leading cause of carcass and organ condemnation. Losses are estimated to exceed R$ 500 million per year, since infected carcasses undergo conditional utilization (chilling or heating) or total condemnation, in addition to limiting export opportunities (Camba & Alves, 2020; Guimarães-Peixoto et al., 2020). According to Rezende et al. (2018), producers experience a 30% reduction in carcass value when subjected to chilling, 50% when subjected to heat treatment, and receive no financial return in cases of total condemnation. Although post-mortem inspection has limited sensitivity, especially in mild infections, it is indispensable. Moreover, these data are essential to estimate economic losses associated with condemnations and to understand the spatial distribution of the disease in different regions (Brasil, 2017; Guimarães-Peixoto et al., 2020).

In this context, the present study aimed to assess the occurrence of bovine cysticercosis in cattle slaughtered under the Federal Inspection Service in Nanuque, Minas Gerais, in 2021 and 2022, estimate the associated productive losses, and analyze the spatial distribution of cases.

## Material and Methods

### Study area

The present study was conducted in a slaughterhouse operating under the Federal Inspection Service (SIF), located in Nanuque, Minas Gerais, from January 2021 to December 2022. All cattle slaughtered at this establishment during the period were considered, totaling 120,011 animals in 2021 and 177,791 in 2022. Animals originated from 70 municipalities, primarily from the Jequitinhonha, Mucuri, and Doce Valley mesoregions.

Spatial distribution of cases was evaluated by mapping the municipalities of origin of slaughtered animals using geographic coordinates obtained from official administrative boundaries. The number of positive animals per municipality was plotted using geographic information system software (ArcGIS Pro v3.1.0) to visualize the spatial distribution of cases within the study region.

### Experimental sampling

The sample size calculation to estimate monthly prevalence was performed using the formula for proportions commonly applied in epidemiological studies):


$$n=\frac{z^{2}\;.\;P\;\left(1-P\right)}{d^{2}}$$ (1)

where:

n = minimum number of animals required

z = 1.96 (95% confidence)

P = expected prevalence (1.03%; Oliveira et al., 2020)

d = standard error (5%)

Thus, the minimum number of carcasses required was 1,567 per month. However, all months evaluated in 2021 and 2022 exceeded this value, with the lowest monthly slaughter total being 6,055 animals, allowing prevalence estimation for all months of the study.

### Post-mortem inspection

Carcass evaluation followed the Industrial and Sanitary Inspection Regulation for Products of Animal Origin (Decree 9.013/17, Art. 185 and its amendments). Carcasses with viable or calcified cysticerci were considered positive. Destination followed legal criteria, in which infection levels were classified as: allowed for consumption (one calcified cysticerci); conditional chilling (≥1 and <8 calcified cysticerci); conditional heat treatment (≥1 and <8 viable cysticerci) and condemnation (≥8 viable or calcified cysticerci).

When cysticerci were detected, affected organs were condemned according to Industrial and Sanitary Inspection Regulation for Products of Animal Origin (Decree 9.013/17, Art. 185 and its amendments) regulations. Although the carcass remained the primary unit for economic loss calculations, the condemnation of viscera resulted in an immediate reduction in carcass commercial value by approximately 10%.

### Economic loss calculations

Economic losses due to bovine cysticercosis were estimated according to RIISPOA (Decree 9.013/2017, Art. 185), considering the carcass as the calculation unit. Depreciation varies according to region and slaughterhouse policies, with reported reductions of up to 65% for viable cysticerci, 30% for chilling, and 50% for heat treatment (Rezende et al., 2018). In the slaughterhouse assessed in this study, penalties adopted were: 30% for carcasses subjected to chilling; 70% for carcasses subjected to heat treatment; 10% referring to condemned viscera (organs such as heart, liver, tongue, esophageal muscle, and head meat) whenever the carcass was sent to the Federal Inspection Department (DIF) for cysticerci counting. Carcass price was estimated based on the annual mean price of finished cattle (CEPEA, 2025), R$ 305.70 in 2021 and R$ 317.80 in 2022, multiplied by the average cold carcass weight of 250 kg.

Formulas used:

1. Full condemnation losses
  $$L_{cond}=N_{cond}\;.\;V_{carc}$$ (2)
2. Initial viscera condemnation (−10%)
  $$V_{after}=0,9\;.\;V_{carc}$$ (3)
3. Losses due to viscera condemnation
  $$L_{visc}=\;N_{visc}\;.\left(0,1\;.\;V_{carc}\right)$$ (4)
4. Losses from carcasses designated to chilling (−30%)
  $$L_{cold}=\;N_{cold}\;.\left(0,3\;.\;V_{after}\right)$$ (5)
5. Losses from carcasses designated to heating (−70%)
  $$L_{heat}=\;N_{heat}\;.\left(0,7\;.\;V_{after}\right)$$ (6)
6. Total loss sum
  $$L_{total}=L_{cond}+\;L_{visc}+\;L_{cold}+\;L_{heat}$$ (7)

L_cond_: Loss by full condemnation; N_cond_: Number of fully condemned carcasses; V_carc_: Mean value of each carcass; V_after_: Value after removal of condemned viscera; N_visc_: Number of carcasses that had their viscera condemned; L_visc_: Loss by viscera condemnation; L_cold_; Loss by cold treatment; N_cold_: Number of carcasses that underwent cold treatment; L_heat_: Loss by heat treatment; N_heat_: Number of carcasses that underwent heat treatment.

## Results

The prevalence of animals presenting viable or calcified cysticerci was 0.44% (531/120,011) in 2021 and 0.24% (419/177,791) in 2022 (Table 1). Monthly prevalence remained relatively stable throughout both years, ranging from 0.34% to 0.92% in 2021 and from 0.13% to 0.37% in 2022. There was a significant difference in prevalence between 2021 and 2022 (χ^2^ = 96.1; p < 0.001).

**Table 1:** Monthly prevalence of slaughtered cattle, number of positive cases, and percentage of bovine cysticercosis in a slaughterhouse under Federal Inspection Service in Minas Gerais, in the years 2021 and 2022.

**Months**	**2021**			**2022**		
	**Slaughtered (n)**	**Cases**	**Prevalence (CI 95%)**	**Slaughtered (n)**	**Cases**	**Prevalence (CI 95%)**
January	10396	35	0.34% (0.23–0.45%)	11541	28	0.24% (0.15–0.33%)
February	8879	50	0.56% (0.41–0.72%)	12936	36	0.28% (0.19–0.37%)
March	7892	30	0.38% (0.24–0.52%)	16210	37	0.23% (0.15–0.30%)
April	9155	35	0.38% (0.26–0.51%)	14081	37	0.26% (0.18–0.35%)
May	10840	38	0.35% (0.24–0.46%)	17250	64	0.37% (0.28–0.46%)
June	11588	35	0.30% (0.20–0.40%)	14994	23	0.15% (0.09–0.22%)
July	12808	45	0.35% (0.25–0.45%)	17248	38	0.22% (0.15–0.29%)
August	12435	56	0.45% (0.33–0.57%)	18000	57	0.32% (0.23–0.40%)
September	6055	56	0.92% (0.68–1.17%)	15750	20	0.13% (0.07–0.18%)
October	10079	41	0.41% (0.28–0.53%)	15667	29	0.19% (0.12–0.25%)
November	10452	53	0.51% (0.37–0.64%)	11959	32	0.27% (0.17–0.36%)
December	9432	57	0.60% (0.45–0.76%)	12155	18	0.15% (0.08–0.22%)
TOTAL	120011	531	0.44% (0.40-0.48%)	177791	419	0.24% (0.18–0.30%)

CI: Confidence interval (95%).

**Figure 1:**
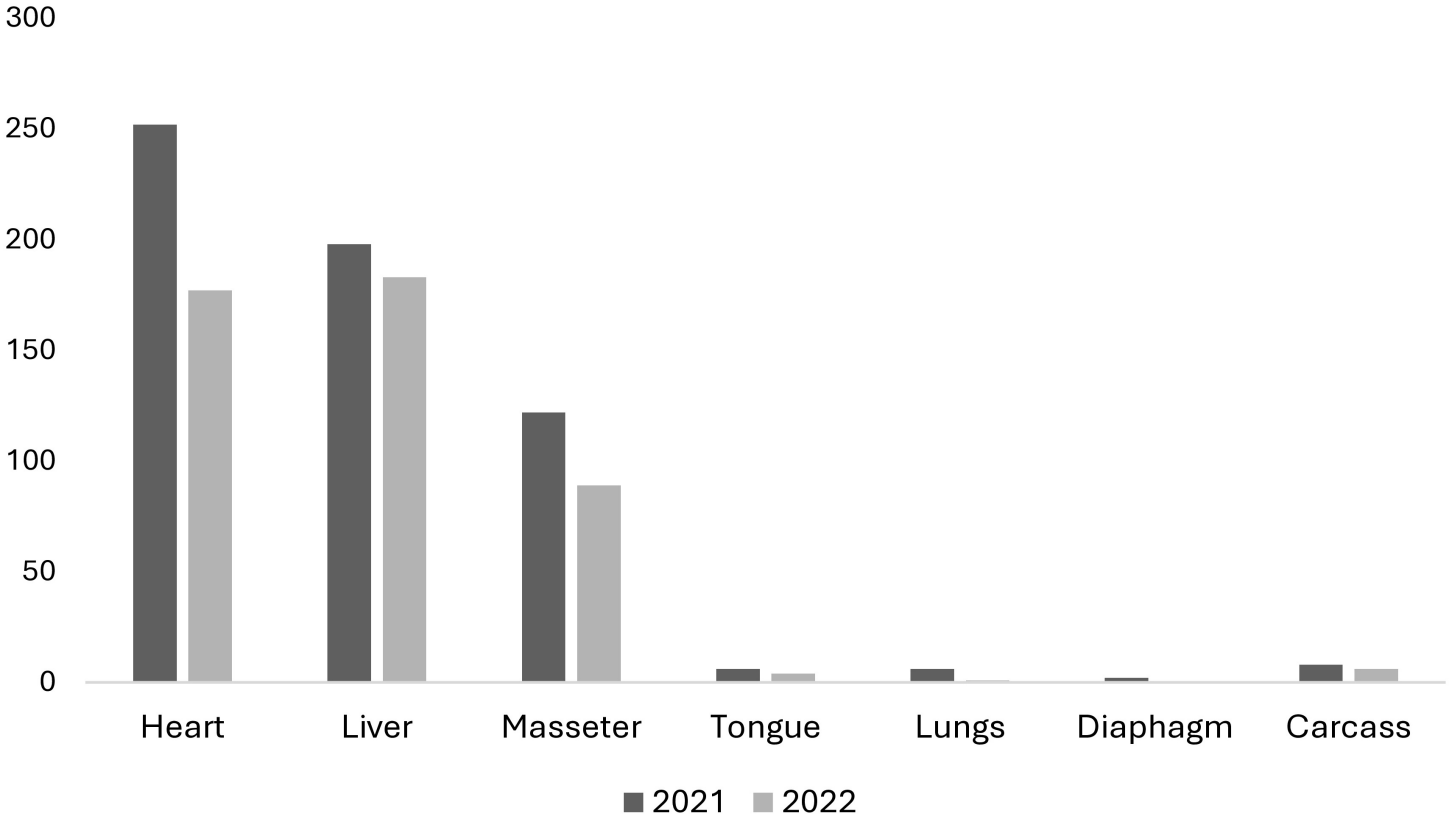
Anatomical distribution of Cysticercus bovis cysticerci in positive bovine carcasses, according to the affected organ, in the years 2021 and 2022.

Cysticerci locations in positive carcasses (n = 950) were most frequent in the heart: 252 cases in 2021 and 177 in 2022, totaling 45.2% (429/950). The liver was the second most affected organ, with 198 cases in 2021 and 183 in 2022, totaling 40.1% (381/950). Cysticerci were found in the masseter muscle in 112 carcasses in 2021 and 89 in 2022 (21.2%, 201/950). Rare occurrences included the tongue (10 cases), lungs (7), diaphragm (2), and carcass musculature (14 cases). This distribution indicates a clear predilection for major internal organs (heart and liver) compared with uncommon locations (Figure 1).

Across the two years, most positive carcasses presented calcified cysticerci (82.8%, 787/950), whereas viable cysticerci accounted for 17.2% (163/950). No carcass with viable cysticerci was released for direct consumption; all were designated for conditional utilization, mainly chilling, followed by heat treatment. Among calcified cysticerci cases, most carcasses were released for consumption (75.2%, 592/787), though a significant portion (24.6%, 194/787) underwent conditional treatment (Table 2).

**Table 2:** Number of bovine carcasses with a positive diagnosis for bovine cysticercosis in the years 2021 and 2022 and their destination in a slaughterhouse under Federal Inspection Service in Minas Gerais.

	**Positive carcasses**	**Carcasses allowed for consumption**	**Chilling treatment**	**Heat treatment**	**Condemned carcasses**
**2021**					
Viable cysticerci	98 (18.5%)	0	64 (50%)	31 (34.8%)	3 (75%)
Calcified cysticerci	433 (81.5%)	310 (100%)	64 (50%)	58 (65.2%)	1 (25%)
TOTAL	531	310	128	89	4
**2022**					
Viable cysticerci	65 (15.5%)	0	38 (51.4%)	25 (41%)	2 (100%)
Calcified cysticerci	354 (84.5%)	282 (100%)	36 (48.6%)	36 (59%)	0
TOTAL	419	282	74	61	2

Estimated economic losses due to carcass devaluation from treatment or disposal totaled R$ 934,056.01 during the two years. In 2021, losses reached R$ 556,229.68, primarily from carcasses subjected to chilling (58.0%; R$ 322,411.52) and heat treatment (27.8%; R$ 154,853.78). In 2022, losses totaled R$ 377,826.33, of which 56.8% (R$ 214,599.85) corresponded to chilling and 27.5% (R$ 103,991.31) to heat treatment. Total condemnations accounted for 1.5% (R$ 14,200.00) of all losses.

**Figure 2:**
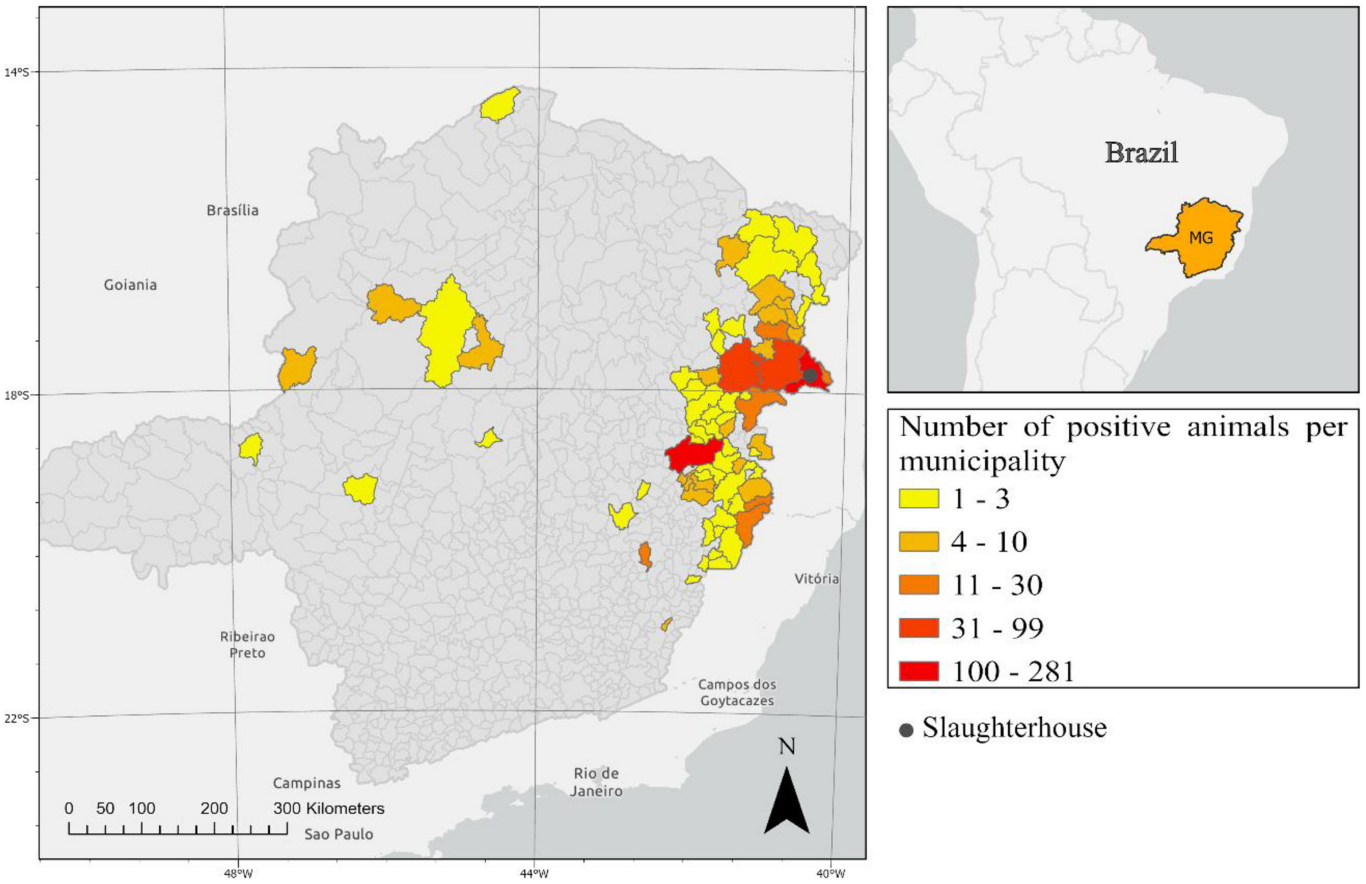
Distribution by municipality of cases of bovine cysticercosis in animals slaughtered between 2021 and 2022 in a slaughterhouse under Federal Inspection Service, in the state of Minas Gerais.

The municipalities of Governador Valadares (176 and 80), Nanuque (169 and 112), and Carlos Chagas (29 and 52) were the main sources of positive animals in 2021 and 2022, respectively, contributing 70.4% and 58.2% of diagnosed cases (Figure 2).

A total of 210 different farms supplied animals to the slaughterhouse over the two years. In Carlos Chagas, 81 positive animals originated from 43 farms, averaging two positive animals per farm. In contrast, Nanuque accounted for 281 cases from only 35 farms, averaging eight positive animals per farm. Notably, a single property in Nanuque accounted for 41.9% (118/281) of the municipality’s reported cases, indicating considerable heterogeneity in the contribution of farms to bovine cysticercosis occurrence.

## Discussion

The total prevalence of bovine cysticercosis observed in this study was 0.32% in 2021–2022. Although a statistically significant difference was observed between years (0.44% and 0.24%; χ^2^ = 96.1; p < 0.001), this result should be interpreted cautiously given the descriptive nature of the dataset and the large sample size. The prevalence value is lower than reports from some Brazilian regions, these values confirm the persistence of the taeniasis–cysticercosis complex as a sanitary and economic challenge for national cattle production. In Espírito Santo, Rossi et al. (2022) reported a prevalence of 0.57% in cattle slaughtered under federal inspection, while Oliveira et al. (2020) found 1.03% prevalence in northern Minas Gerais. Similarly, Aquino et al. (2017) reported a prevalence of 0.53% in cattle slaughtered in the state of Goiás using large-scale slaughterhouse inspection data. A broader analysis based on slaughterhouse records from approximately 22 million bovine carcasses slaughtered between 2018 and 2020 across ten Brazilian states from the North, Northeast, Southeast and Center-West regions reported an overall prevalence of 0.09%, highlighting substantial spatial variation in bovine cysticercosis occurrence in Brazil (Dias et al., 2025). Across the Americas, bovine cysticercosis prevalence varies widely, ranging from 0.1% to 19%, depending on country and region, from Canada to Argentina (Braae et al., 2018).

In the present study, all animals evaluated originated exclusively from properties that slaughter under the Federal Inspection System (SIF), which adopts stricter criteria to ensure export compliance (RIISPOA, Decree 9.013/2017, Art. 185). This likely contributed to the reduced prevalence compared with other studies, since export-oriented farms tend to have higher technological development, better management practices, and stronger sanitary control.

Transmission is inherently linked to humans as definitive hosts of *T. saginata*, who serve as the primary infection source for cattle. Contamination occurs mainly through improper disposal of human feces or the use of untreated effluents for irrigation, resulting in deposition of eggs onto pastures and water sources consumed by cattle (Bucur et al., 2019; Elbarbary et al., 2025).

Monthly analysis showed no clear seasonal trend. Although other cattle parasites often vary with rainfall patterns or pasture management (McFarland et al., 2022), the proportion of positive carcasses remained stable throughout the study period. This reinforces the hypothesis that transmission is associated with continuous factors such as inadequate sanitation and the presence of human carriers, rather than environmental fluctuations (Bucur et al., 2019). The persistence of low yet constant rates suggests ongoing year-round risk.

Economically, the findings were also significant. Even a single cysticerci, viable or calcified, results in immediate condemnation of viscera, reducing approximately 10% of carcass value. According to legal criteria (RIISPOA, Decree 9.013/2017, Art. 185), carcasses may then undergo conditional utilization: chilling (30% reduction) or heat treatment (70% reduction). In this study, 128 carcasses were chilled and 89 heat-treated in 2021; in 2022, 74 were chilled and 61 heat-treated.

Despite the relatively low overall prevalence (0.32%), cumulative losses exceeded R$ 900,000 across two years. Similar economic impacts have been reported in large-scale retrospective analyses based on slaughterhouse inspection data. In the state of Goiás, Aquino et al. (2017) estimated losses exceeding US$ 9 million associated with bovine cysticercosis during an eight-year period. This figure is likely underestimated since a significant portion of carcasses slaughtered under federal inspection are destined for export markets, where beef has greater commercial value. Thus, economic impact extends beyond direct producer losses, also affecting Brazil’s international competitiveness in the beef trade.

Regarding anatomical distribution, most cysticerci were observed in the heart and liver, differing from other reports indicating the tongue and masseter muscles as primary predilection sites (Rezende et al., 2018; Fesseha & Asefa, 2023). However, similar findings have been reported in other Brazilian studies. de Souza et al. (2025b) identified cysticerci in both the liver and masseter muscles during post-mortem inspection of cattle slaughtered in Northeastern Brazil. Conversely, in a study from Mexico, González et al. (2015) found the heart and liver to be the most common sites. This divergence indicates no universally preferred organ; instead, anatomical distribution may vary depending on epidemiological context, cattle population, and post-mortem inspection sensitivity (Elbarbary et al., 2025).

More frequent detection in internal organs may reflect rigorous inspection practices under SIF protocols, which mandate incisions in these sites (RIISPOA, Decree 9.013/2017), as well as potential regional factors not yet fully understood. Such discrepancies highlight the need for systematic evaluation of all required organs, given wide variability reported across studies and geographic regions.

The spatial distribution of cases indicated a higher concentration of positive animals originating from municipalities located in eastern Minas Gerais, particularly Nanuque, Governador Valadares, and Carlos Chagas. These municipalities either surround or closely neighbor the slaughterhouse, possibly leading to greater case numbers due to higher animal supply. They lie within the Jequitinhonha, Mucuri, and Doce river valleys, which are historically marked by social inequities and inadequate sanitation (Gomes et al., 2023). Similar spatial heterogeneity has been reported in other Brazilian regions by Ferreira et al. (2014), which identified higher occurrence of bovine cysticercosis in areas with lower human development indices and intense agricultural activity, suggesting that human presence and sanitation conditions may contribute to environmental contamination with *T. saginata* eggs.

According to the 2022 IBGE census (IBGE, 2022), only 59.5% and 69.7% of the populations of Jequitinhonha and Carlos Chagas, respectively, are connected to a sewage network, and more than a quarter still rely on rudimentary pits (25.6% and 26.9%). In Nanuque, although the percentage is lower, it remains significant (7.7%). In contrast, Governador Valadares has high sanitation coverage (94.4%). Nevertheless, Nanuque and Governador Valadares presented the highest number of cases. This apparent contradiction may be explained by river dynamics, in which both municipalities receive water from rivers flowing through cities with precarious sanitation, such as Jequitinhonha and Carlos Chagas.

Fecal contamination of the Doce, Mucuri, and Jequitinhonha rivers was demonstrated by Gomes et al. (2023) in a study on schistosomiasis mortality in Minas Gerais. They showed that freshwater bodies in these regions contained significant fecal contamination. The potential discharge of effluents containing *T. saginata* eggs or proglottids into these rivers may affect downstream water quality even in areas with robust sanitation. This suggests that bovine cysticercosis transmission may depend not only on local infrastructure but also on environmental vulnerability imposed by regional hydrographic dynamics (Duarte et al., 2016).

An important limitation of this study is that it was conducted exclusively in a slaughterhouse under federal inspection. Although the region has one such establishment, factors such as distance, low technological development of farms, structural limitations, and logistical costs may lead some producers to choose clandestine slaughter (Vale et al., 2019).

This dynamic may artificially reduce prevalence observed in SIF facilities, since animals from more vulnerable production systems are less likely to enter the formal inspection circuit (Vale et al., 2019). Thus, the prevalence found in this study may be underestimated when compared with neighboring regions in Espírito Santo (Rossi et al., 2022) and northern Minas Gerais (Oliveira et al., 2020), where higher rates (0.57% and 1.07%) were reported. This reinforces the need to consider regional production and sanitary contexts when interpreting prevalence derived exclusively from federally inspected units.

A limitation of this study is that detailed individual-level data were available only for positive animals, as records were obtained from slaughterhouse inspection services. Therefore, analytical epidemiological approaches, such as risk factor analysis, were not feasible.

## Conclusion

Bovine cysticercosis showed prevalence of 0.44% in 2021 and 0.24% in 2022 in the Jequitinhonha mesoregion and the Mucuri and Doce river valleys, resulting in economic losses exceeding R$ 900,000. Despite slaughter occurring under strict and technically advanced Federal Inspection Service conditions, the disease persists as a sanitary and economic barrier, and spatial analysis highlighted the influence of environmental vulnerability within regional watersheds in sustaining disease transmission.

## Data Availability

The data supporting the findings of this study are available upon request from the corresponding author.
